# (2,2′-Bipyridyl-κ^2^ *N*,*N*′)chlorido(dl-threoninato-κ^2^ *N*,*O* ^1^)copper(II) monohydrate

**DOI:** 10.1107/S1600536811002583

**Published:** 2011-01-26

**Authors:** Yi-Han Tan, Siang-Guan Teoh, Mohd Mustaqim Rosli, Hoong-Kun Fun

**Affiliations:** a School of Chemical Sciences, Universiti Sains Malaysia, 11800 USM, Penang, Malaysia; b X-ray Crystallography Unit, School of Physics, Universiti Sains Malaysia, 11800 USM, Penang, Malaysia

## Abstract

In the title compound, [Cu(C_4_H_8_NO_3_)Cl(C_10_H_8_N_2_)]·H_2_O, the Cu^II^ atom is in a slightly distorted square-pyramidal coordination geometry with the basal plane defined by the two N atoms of the bipyridine ligand and the N and O atoms from the threoninate ion and the apical site occupied by the Cl atom. In the crystal, inter­molecular O—H⋯O, N—H⋯O, O—H⋯Cl, C—H⋯O and C—H⋯Cl inter­actions link the mol­ecules into a three-dimensional network. A π–π inter­action with a centroid–centroid distance of 3.461 (1) Å is also present.

## Related literature

For background to superoxide dismutase activity, see: Kumar & Arunachalam (2007 ▶); Patel *et al.* (2006 ▶); Rao *et al.* (2007 ▶); Zhang *et al.* (2004 ▶). For a related structure, see: Tan *et al.* (2010 ▶).
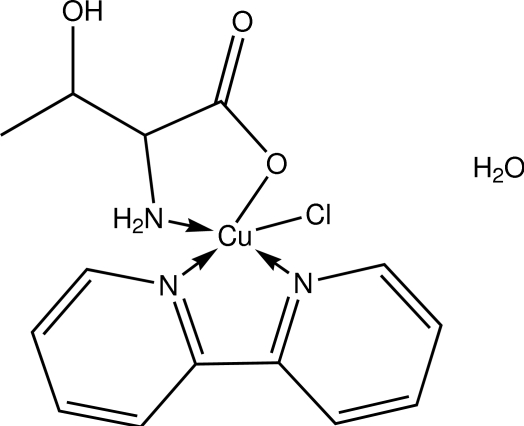


## Experimental

### 

#### Crystal data

[Cu(C_4_H_8_NO_3_)Cl(C_10_H_8_N_2_)]·H_2_O
*M* *_r_* = 391.30
Monoclinic, 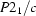

*a* = 7.4825 (1) Å
*b* = 12.0378 (2) Å
*c* = 18.2083 (3) Å
β = 99.097 (1)°
*V* = 1619.44 (4) Å^3^
*Z* = 4
Mo *K*α radiation
μ = 1.54 mm^−1^
*T* = 297 K
0.39 × 0.33 × 0.10 mm

#### Data collection

Bruker SMART APEXII CCD area-detector diffractometer
Absorption correction: multi-scan (*SADABS*; Bruker, 2009 ▶) *T*_min_ = 0.585, *T*_max_ = 0.864
14209 measured reflections
3767 independent reflections
3262 reflections with *I* > 2σ(*I*)
*R*_int_ = 0.026

#### Refinement

*R*[*F* ^2^ > 2σ(*F* ^2^)] = 0.033
*wR*(*F* ^2^) = 0.086
*S* = 1.05
3767 reflections
221 parameters
H atoms treated by a mixture of independent and constrained refinement
Δρ_max_ = 0.61 e Å^−3^
Δρ_min_ = −0.33 e Å^−3^

### 

Data collection: *APEX2* (Bruker, 2009 ▶); cell refinement: *SAINT* (Bruker, 2009 ▶); data reduction: *SAINT*; program(s) used to solve structure: *SHELXTL* (Sheldrick, 2008 ▶); program(s) used to refine structure: *SHELXTL*; molecular graphics: *SHELXTL*; software used to prepare material for publication: *SHELXTL* and *PLATON* (Spek, 2009 ▶).

## Supplementary Material

Crystal structure: contains datablocks global, I. DOI: 10.1107/S1600536811002583/is2663sup1.cif

Structure factors: contains datablocks I. DOI: 10.1107/S1600536811002583/is2663Isup2.hkl

Additional supplementary materials:  crystallographic information; 3D view; checkCIF report

## Figures and Tables

**Table 1:** Hydrogen-bond geometry (Å, °)

*D*—H⋯*A*	*D*—H	H⋯*A*	*D*⋯*A*	*D*—H⋯*A*
O1*W*—H1*W*1⋯O3^i^	0.77	2.12	2.875 (3)	168
O1*W*—H2*W*1⋯Cl1^ii^	0.84	2.38	3.213 (2)	170
N3—H1*N*3⋯O2^iii^	0.85 (3)	2.20 (2)	2.978 (3)	152 (3)
N3—H2*N*3⋯O1*W*	0.80 (3)	2.26 (3)	3.051 (3)	167 (3)
O3—H1*O*3⋯Cl1^iv^	0.88 (4)	2.29 (4)	3.118 (2)	156 (3)
C3—H3*A*⋯O2^v^	0.93	2.55	3.219 (4)	130
C4—H4*A*⋯Cl1^vi^	0.93	2.67	3.555 (2)	160

Symmetry codes: (i) 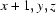; (ii) 
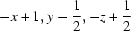
; (iii) 
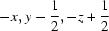
; (iv) 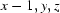; (v) 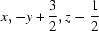; (vi) 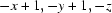.

**Fig. 1.**
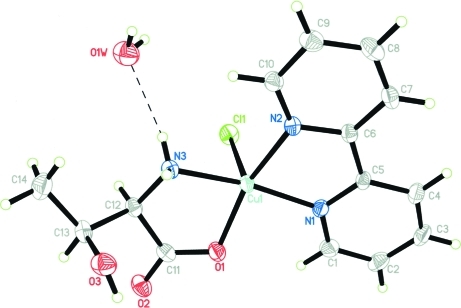
The molecular structure, showing 30% probability displacement ellipsoids and the atom-numbering scheme. Hydrogen atoms are shown as spheres of arbitrary radius.

**Fig. 2.**
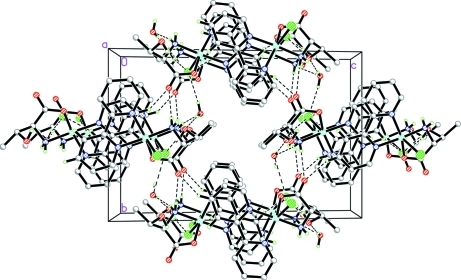
The crystal packing of (I) viewed along the c axis. Dashed lines indicate hydrogen bonds. H atoms not involved in the hydrogen bond interactions have been omitted for clarity.

## supplementary crystallographic information

### Comment

Some copper complexes with amino acid ligands have been studied for the behavior of copper enzymes. Several reports showed that copper complexes of amino acids exhibit effective antitumor and artificial nuclease activities as they cleave DNA efficiently by oxidative and hydrolytic pathways. Copper(II) complexes play an important role in naturally occurring biological systems and act as pharmaceutical agents. Copper complexes containing polypyridyl ligands have received great attention as they exhibit a variety of biological properties such as antimycobaterial, anticandida, antitumor and antimicrobial activities. Mixed ligands copper complexes were reported to exhibit superoxide dismutase activity (Patel *et al.*, 2006; Zhang *et al.*, 2004; Kumar & Arunachalam, 2007; Rao *et al.*, 2007; Tan *et al.*, 2010). In the title compound, DL-threonine has been selected as it is a bio-essential amino acid.

All parameters in compound, (Fig. 1), are within normal range. The Cu^II^ is in a slightly distorted square-pyramidal coordination geometry, with the basal plane being defined by N1 and N2 atoms from the bipyridine group and N3 and O1 atoms from the threoninato group. The apical position is occupied by atom Cl1. The N3—H2N3···O1W interaction linked the water molecule with the main compound.

In the crystal structure (Fig. 2), intermolecular O1W—H1W1···O3^i^, O1W—H2W1···Cl1^ii^, N3—H1N3···O2^iii^, O3—H1O3···Cl1^iv^, C3—H3A···O2^v^ and C4—H4A···Cl1^vi^ (Table 1) interactions  link the molecules into a three-dimensional network. The crystal packing is further stabilized by a π–π stacking interaction with *Cg*1···*Cg*2 distance of 3.461 (1) Å (*Cg*1 = centroid of Cu1/N1/N2/C5/C6 and *Cg*2 = centroid of N1/C1–C5).

### Experimental

To an ethanolic solution (10 mL) of copper(II) chloride dihydrate (0.1708 g, 1 mmol), an ethanolic solution (10 mL) of DL-threonine (0.1191 g, 1 mmol) as well as an ethanolic solution (10 mL) of 2,2'-bipyridyl (0.1561 g, 1 mmol) were added. The pH of the resulting solution was then adjusted to pH 8 by adding a few drops of NaOH aqueous solution. The blue precipitate formed was filtered and single crystals suitable for X-ray diffraction were obtained by recrystallization of the complex.

### Refinement

The water molecule's hydrogen atoms were located in a difference map and refined using a riding model, with *U*_iso_(H) = 1.5*U*_eq_(O). H atoms attached to N and other O atoms were located in a difference Fourier map and were freely refined. The remaining H atoms were positioned geometrically [C—H = 0.93 to 0.98 Å] and refined using a riding model, with *U*_iso_(H) = 1.2 or 1.5 *U*_eq_(C). A rotating-group model was applied for the methyl group.

### Crystal data

**Table:**

[Cu(C_4_H_8_NO_3_)Cl(C_10_H_8_N_2_)]·H_2_O	*F*(000) = 804
*M**_r_* = 391.30	*D*_x_ = 1.605 Mg m^−^^3^
Monoclinic, *P*2_1_/*c*	Mo *K*α radiation, λ = 0.71073 Å
Hall symbol: -P 2ybc	Cell parameters from 6390 reflections
*a* = 7.4825 (1) Å	θ = 2.3–27.7°
*b* = 12.0378 (2) Å	µ = 1.54 mm^−^^1^
*c* = 18.2083 (3) Å	*T* = 297 K
β = 99.097 (1)°	Block, blue
*V* = 1619.44 (4) Å^3^	0.39 × 0.33 × 0.10 mm
*Z* = 4	

### Data collection

**Table:**

Bruker SMART APEXII CCD area-detector diffractometer	3767 independent reflections
Radiation source: fine-focus sealed tube	3262 reflections with *I* > 2σ(*I*)
graphite	*R*_int_ = 0.026
φ and ω scans	θ_max_ = 27.7°, θ_min_ = 2.0°
Absorption correction: multi-scan (*SADABS*; Bruker, 2009)	*h* = −9→9
*T*_min_ = 0.585, *T*_max_ = 0.864	*k* = −15→15
14209 measured reflections	*l* = −22→23

### Refinement

**Table:**

Refinement on *F*^2^	Primary atom site location: structure-invariant direct methods
Least-squares matrix: full	Secondary atom site location: difference Fourier map
*R*[*F*^2^ > 2σ(*F*^2^)] = 0.033	Hydrogen site location: inferred from neighbouring sites
*wR*(*F*^2^) = 0.086	H atoms treated by a mixture of independent and constrained refinement
*S* = 1.05	*w* = 1/[σ^2^(*F*_o_^2^) + (0.0357*P*)^2^ + 1.3397*P*] where *P* = (*F*_o_^2^ + 2*F*_c_^2^)/3
3767 reflections	(Δ/σ)_max_ = 0.001
221 parameters	Δρ_max_ = 0.61 e Å^−^^3^
0 restraints	Δρ_min_ = −0.33 e Å^−^^3^

### Special details

**Table:**

Geometry. All esds (except the esd in the dihedral angle between two l.s. planes) are estimated using the full covariance matrix. The cell esds are taken into account individually in the estimation of esds in distances, angles and torsion angles; correlations between esds in cell parameters are only used when they are defined by crystal symmetry. An approximate (isotropic) treatment of cell esds is used for estimating esds involving l.s. planes.
Refinement. Refinement of F^2^ against ALL reflections. The weighted R-factor wR and goodness of fit S are based on F^2^, conventional R-factors R are based on F, with F set to zero for negative F^2^. The threshold expression of F^2^ > 2sigma(F^2^) is used only for calculating R-factors(gt) etc. and is not relevant to the choice of reflections for refinement. R-factors based on F^2^ are statistically about twice as large as those based on F, and R- factors based on ALL data will be even larger.

### Fractional atomic coordinates and isotropic or equivalent isotropic displacement parameters (Å^2^)

**Table:**

	*x*	*y*	*z*	*U*_iso_*/*U*_eq_	
Cu1	0.21099 (4)	0.51646 (2)	0.150342 (15)	0.03011 (9)	
Cl1	0.50867 (8)	0.61287 (6)	0.20278 (4)	0.04414 (16)	
O1	0.0535 (2)	0.63696 (14)	0.17196 (9)	0.0410 (4)	
O2	−0.0305 (3)	0.72701 (15)	0.26746 (11)	0.0485 (5)	
O3	−0.1859 (3)	0.46636 (18)	0.29026 (12)	0.0496 (5)	
N1	0.1999 (2)	0.57032 (16)	0.04640 (10)	0.0313 (4)	
N2	0.3031 (2)	0.37847 (15)	0.10406 (10)	0.0311 (4)	
N3	0.1738 (3)	0.45802 (17)	0.24897 (11)	0.0323 (4)	
C1	0.1465 (3)	0.6719 (2)	0.02282 (15)	0.0426 (6)	
H1A	0.1111	0.7221	0.0565	0.051*	
C2	0.1426 (4)	0.7044 (2)	−0.04996 (15)	0.0489 (7)	
H2A	0.1038	0.7753	−0.0652	0.059*	
C3	0.1965 (4)	0.6308 (3)	−0.09978 (14)	0.0471 (6)	
H3A	0.1946	0.6513	−0.1491	0.056*	
C4	0.2534 (3)	0.5266 (2)	−0.07581 (13)	0.0411 (6)	
H4A	0.2920	0.4760	−0.1086	0.049*	
C5	0.2525 (3)	0.49774 (19)	−0.00186 (12)	0.0301 (5)	
C6	0.3081 (3)	0.38790 (19)	0.03022 (12)	0.0310 (5)	
C7	0.3610 (3)	0.3007 (2)	−0.01053 (14)	0.0414 (6)	
H7A	0.3630	0.3085	−0.0612	0.050*	
C8	0.4109 (4)	0.2015 (2)	0.02508 (17)	0.0484 (6)	
H8A	0.4470	0.1417	−0.0014	0.058*	
C9	0.4068 (4)	0.1920 (2)	0.10013 (16)	0.0446 (6)	
H9A	0.4409	0.1261	0.1251	0.054*	
C10	0.3511 (3)	0.2820 (2)	0.13779 (14)	0.0381 (5)	
H10A	0.3469	0.2752	0.1884	0.046*	
C11	0.0373 (3)	0.64663 (19)	0.24007 (14)	0.0351 (5)	
C12	0.1085 (3)	0.5511 (2)	0.29117 (13)	0.0339 (5)	
H12A	0.2128	0.5791	0.3256	0.041*	
C13	−0.0346 (4)	0.5158 (2)	0.33788 (14)	0.0407 (6)	
H13A	−0.0767	0.5817	0.3617	0.049*	
C14	0.0338 (5)	0.4324 (3)	0.39667 (17)	0.0614 (8)	
H14A	−0.0561	0.4205	0.4281	0.092*	
H14B	0.1429	0.4596	0.4260	0.092*	
H14C	0.0584	0.3635	0.3736	0.092*	
O1W	0.5123 (3)	0.36924 (19)	0.34690 (13)	0.0643 (6)	
H1W1	0.6010	0.3869	0.3342	0.096*	
H2W1	0.5185	0.3006	0.3382	0.096*	
H1N3	0.098 (4)	0.405 (2)	0.2438 (14)	0.033 (7)*	
H2N3	0.264 (4)	0.428 (2)	0.2692 (16)	0.042 (8)*	
H1O3	−0.246 (5)	0.523 (3)	0.268 (2)	0.064 (11)*	

### Atomic displacement parameters (Å^2^)

**Table:**

	*U*^11^	*U*^22^	*U*^33^	*U*^12^	*U*^13^	*U*^23^
Cu1	0.03619 (16)	0.03037 (15)	0.02525 (15)	0.00142 (11)	0.00949 (11)	−0.00088 (11)
Cl1	0.0391 (3)	0.0492 (4)	0.0433 (3)	−0.0077 (3)	0.0043 (2)	−0.0089 (3)
O1	0.0500 (10)	0.0394 (9)	0.0360 (9)	0.0098 (8)	0.0146 (8)	0.0018 (7)
O2	0.0594 (11)	0.0371 (9)	0.0529 (11)	0.0046 (8)	0.0214 (9)	−0.0086 (8)
O3	0.0383 (10)	0.0506 (12)	0.0613 (13)	−0.0045 (9)	0.0120 (9)	0.0059 (10)
N1	0.0314 (9)	0.0358 (10)	0.0264 (9)	−0.0004 (8)	0.0038 (7)	0.0018 (8)
N2	0.0334 (9)	0.0323 (9)	0.0284 (9)	−0.0017 (8)	0.0074 (7)	0.0000 (8)
N3	0.0340 (10)	0.0322 (10)	0.0318 (10)	−0.0010 (9)	0.0087 (8)	0.0000 (8)
C1	0.0444 (13)	0.0397 (13)	0.0442 (14)	0.0064 (11)	0.0084 (11)	0.0057 (11)
C2	0.0480 (15)	0.0513 (16)	0.0450 (15)	0.0035 (12)	0.0002 (12)	0.0189 (13)
C3	0.0434 (14)	0.0672 (18)	0.0286 (12)	−0.0052 (13)	0.0000 (10)	0.0143 (12)
C4	0.0388 (12)	0.0556 (16)	0.0283 (12)	−0.0054 (11)	0.0037 (10)	−0.0008 (11)
C5	0.0273 (10)	0.0385 (12)	0.0239 (10)	−0.0059 (9)	0.0025 (8)	−0.0022 (9)
C6	0.0273 (10)	0.0367 (12)	0.0297 (11)	−0.0046 (9)	0.0069 (8)	−0.0040 (9)
C7	0.0449 (13)	0.0467 (14)	0.0339 (13)	−0.0029 (11)	0.0105 (10)	−0.0108 (11)
C8	0.0500 (15)	0.0401 (14)	0.0576 (17)	0.0005 (12)	0.0157 (13)	−0.0156 (13)
C9	0.0458 (14)	0.0355 (13)	0.0540 (16)	0.0019 (11)	0.0127 (12)	0.0035 (12)
C10	0.0411 (12)	0.0370 (12)	0.0379 (13)	0.0020 (10)	0.0117 (10)	0.0037 (10)
C11	0.0320 (11)	0.0324 (11)	0.0426 (13)	−0.0046 (9)	0.0107 (10)	−0.0066 (10)
C12	0.0337 (11)	0.0369 (12)	0.0320 (12)	−0.0043 (9)	0.0081 (9)	−0.0071 (10)
C13	0.0483 (14)	0.0429 (13)	0.0333 (13)	−0.0021 (11)	0.0144 (11)	−0.0018 (10)
C14	0.077 (2)	0.0639 (19)	0.0447 (17)	0.0044 (17)	0.0156 (15)	0.0145 (15)
O1W	0.0566 (12)	0.0612 (13)	0.0791 (16)	−0.0010 (10)	0.0234 (11)	0.0110 (12)

### Geometric parameters (Å, °)

**Table:**

Cu1—O1	1.9477 (17)	C4—C5	1.392 (3)
Cu1—N3	1.989 (2)	C4—H4A	0.9300
Cu1—N1	1.9898 (18)	C5—C6	1.479 (3)
Cu1—N2	2.0317 (19)	C6—C7	1.379 (3)
Cu1—Cl1	2.5588 (7)	C7—C8	1.382 (4)
O1—C11	1.270 (3)	C7—H7A	0.9300
O2—C11	1.234 (3)	C8—C9	1.376 (4)
O3—C13	1.441 (3)	C8—H8A	0.9300
O3—H1O3	0.88 (4)	C9—C10	1.381 (4)
N1—C1	1.336 (3)	C9—H9A	0.9300
N1—C5	1.342 (3)	C10—H10A	0.9300
N2—C10	1.336 (3)	C11—C12	1.522 (3)
N2—C6	1.356 (3)	C12—C13	1.529 (3)
N3—C12	1.484 (3)	C12—H12A	0.9800
N3—H1N3	0.85 (3)	C13—C14	1.498 (4)
N3—H2N3	0.80 (3)	C13—H13A	0.9800
C1—C2	1.377 (4)	C14—H14A	0.9600
C1—H1A	0.9300	C14—H14B	0.9600
C2—C3	1.373 (4)	C14—H14C	0.9600
C2—H2A	0.9300	O1W—H1W1	0.7668
C3—C4	1.373 (4)	O1W—H2W1	0.8436
C3—H3A	0.9300		
			
O1—Cu1—N3	84.58 (8)	C4—C5—C6	124.1 (2)
O1—Cu1—N1	90.79 (7)	N2—C6—C7	121.7 (2)
N3—Cu1—N1	169.53 (9)	N2—C6—C5	114.61 (19)
O1—Cu1—N2	161.51 (8)	C7—C6—C5	123.7 (2)
N3—Cu1—N2	100.98 (8)	C6—C7—C8	119.0 (2)
N1—Cu1—N2	80.67 (8)	C6—C7—H7A	120.5
O1—Cu1—Cl1	96.05 (6)	C8—C7—H7A	120.5
N3—Cu1—Cl1	93.51 (7)	C9—C8—C7	119.4 (2)
N1—Cu1—Cl1	96.32 (6)	C9—C8—H8A	120.3
N2—Cu1—Cl1	101.14 (5)	C7—C8—H8A	120.3
C11—O1—Cu1	114.81 (15)	C8—C9—C10	118.9 (2)
C13—O3—H1O3	105 (2)	C8—C9—H9A	120.5
C1—N1—C5	119.3 (2)	C10—C9—H9A	120.5
C1—N1—Cu1	124.71 (17)	N2—C10—C9	122.3 (2)
C5—N1—Cu1	116.00 (15)	N2—C10—H10A	118.9
C10—N2—C6	118.8 (2)	C9—C10—H10A	118.9
C10—N2—Cu1	127.17 (16)	O2—C11—O1	125.1 (2)
C6—N2—Cu1	114.03 (15)	O2—C11—C12	118.2 (2)
C12—N3—Cu1	107.76 (15)	O1—C11—C12	116.7 (2)
C12—N3—H1N3	110.7 (17)	N3—C12—C11	111.47 (19)
Cu1—N3—H1N3	110.6 (17)	N3—C12—C13	113.29 (19)
C12—N3—H2N3	115 (2)	C11—C12—C13	109.98 (19)
Cu1—N3—H2N3	110 (2)	N3—C12—H12A	107.3
H1N3—N3—H2N3	102 (3)	C11—C12—H12A	107.3
N1—C1—C2	121.9 (3)	C13—C12—H12A	107.3
N1—C1—H1A	119.0	O3—C13—C14	107.4 (2)
C2—C1—H1A	119.0	O3—C13—C12	109.4 (2)
C3—C2—C1	119.2 (3)	C14—C13—C12	113.2 (2)
C3—C2—H2A	120.4	O3—C13—H13A	108.9
C1—C2—H2A	120.4	C14—C13—H13A	108.9
C4—C3—C2	119.2 (2)	C12—C13—H13A	108.9
C4—C3—H3A	120.4	C13—C14—H14A	109.5
C2—C3—H3A	120.4	C13—C14—H14B	109.5
C3—C4—C5	119.1 (2)	H14A—C14—H14B	109.5
C3—C4—H4A	120.4	C13—C14—H14C	109.5
C5—C4—H4A	120.4	H14A—C14—H14C	109.5
N1—C5—C4	121.2 (2)	H14B—C14—H14C	109.5
N1—C5—C6	114.65 (19)	H1W1—O1W—H2W1	97.9
			
N3—Cu1—O1—C11	18.27 (17)	C1—N1—C5—C6	−179.8 (2)
N1—Cu1—O1—C11	−171.14 (17)	Cu1—N1—C5—C6	0.9 (2)
N2—Cu1—O1—C11	126.9 (2)	C3—C4—C5—N1	−1.0 (4)
Cl1—Cu1—O1—C11	−74.70 (16)	C3—C4—C5—C6	179.2 (2)
O1—Cu1—N1—C1	17.4 (2)	C10—N2—C6—C7	0.1 (3)
N3—Cu1—N1—C1	81.0 (5)	Cu1—N2—C6—C7	−177.65 (17)
N2—Cu1—N1—C1	−179.0 (2)	C10—N2—C6—C5	180.0 (2)
Cl1—Cu1—N1—C1	−78.73 (19)	Cu1—N2—C6—C5	2.2 (2)
O1—Cu1—N1—C5	−163.29 (16)	N1—C5—C6—N2	−2.0 (3)
N3—Cu1—N1—C5	−99.7 (5)	C4—C5—C6—N2	177.8 (2)
N2—Cu1—N1—C5	0.23 (15)	N1—C5—C6—C7	177.8 (2)
Cl1—Cu1—N1—C5	100.54 (15)	C4—C5—C6—C7	−2.4 (4)
O1—Cu1—N2—C10	−115.5 (3)	N2—C6—C7—C8	−0.3 (4)
N3—Cu1—N2—C10	−9.4 (2)	C5—C6—C7—C8	179.8 (2)
N1—Cu1—N2—C10	−178.9 (2)	C6—C7—C8—C9	0.0 (4)
Cl1—Cu1—N2—C10	86.41 (19)	C7—C8—C9—C10	0.6 (4)
O1—Cu1—N2—C6	62.1 (3)	C6—N2—C10—C9	0.5 (4)
N3—Cu1—N2—C6	168.12 (15)	Cu1—N2—C10—C9	177.91 (18)
N1—Cu1—N2—C6	−1.37 (15)	C8—C9—C10—N2	−0.8 (4)
Cl1—Cu1—N2—C6	−96.04 (15)	Cu1—O1—C11—O2	167.49 (19)
O1—Cu1—N3—C12	−18.87 (15)	Cu1—O1—C11—C12	−12.2 (3)
N1—Cu1—N3—C12	−83.0 (5)	Cu1—N3—C12—C11	17.4 (2)
N2—Cu1—N3—C12	178.96 (15)	Cu1—N3—C12—C13	142.08 (18)
Cl1—Cu1—N3—C12	76.88 (15)	O2—C11—C12—N3	176.1 (2)
C5—N1—C1—C2	0.5 (4)	O1—C11—C12—N3	−4.2 (3)
Cu1—N1—C1—C2	179.73 (19)	O2—C11—C12—C13	49.6 (3)
N1—C1—C2—C3	−0.6 (4)	O1—C11—C12—C13	−130.7 (2)
C1—C2—C3—C4	−0.1 (4)	N3—C12—C13—O3	−57.7 (3)
C2—C3—C4—C5	0.9 (4)	C11—C12—C13—O3	67.8 (3)
C1—N1—C5—C4	0.4 (3)	N3—C12—C13—C14	62.1 (3)
Cu1—N1—C5—C4	−178.95 (17)	C11—C12—C13—C14	−172.4 (2)

### Hydrogen-bond geometry (Å, °)

**Table:**

*D*—H···*A*	*D*—H	H···*A*	*D*···*A*	*D*—H···*A*
O1W—H1W1···O3^i^	0.77	2.12	2.875 (3)	168
O1W—H2W1···Cl1^ii^	0.84	2.38	3.213 (2)	170
N3—H1N3···O2^iii^	0.85 (3)	2.20 (2)	2.978 (3)	152 (3)
N3—H2N3···O1W	0.80 (3)	2.26 (3)	3.051 (3)	167 (3)
O3—H1O3···Cl1^iv^	0.88 (4)	2.29 (4)	3.118 (2)	156 (3)
C3—H3A···O2^v^	0.93	2.55	3.219 (4)	130
C4—H4A···Cl1^vi^	0.93	2.67	3.555 (2)	160

Symmetry codes: (i) *x*+1, *y*, *z*; (ii) −*x*+1, *y*−1/2, −*z*+1/2; (iii) −*x*, *y*−1/2, −*z*+1/2; (iv) *x*−1, *y*, *z*; (v) *x*, −*y*+3/2, *z*−1/2; (vi) −*x*+1, −*y*+1, −*z*.
